# Role of immune-inflammatory biomarkers and their derived ratio in predicting COVID-19 severity and mortality

**DOI:** 10.1038/s41598-025-24173-7

**Published:** 2025-11-07

**Authors:** Sara I. Taha, Shaimaa H. Fouad, Eman M. El-Sehsah, Mahmoud Mokhtar Mohamed, Azza Omran, Marwa Hamdy, Dalia Hussein Helmy Elsheikh, Basim Othman, Raed A. Alharbi, Rasha Ahmed Ghorab

**Affiliations:** 1 Department of Clinical Pathology, Faculty of Medicine, Ain-Shams University, Cairo, Egypt; 2 Department of Internal Medicine/Allergy and Clinical Immunology, Faculty of Medicine, Ain-Shams University, Cairo, Egypt; 3 Department of Medical Microbiology and Immunology, Mansoura Faculty of Medicine, Mansoura, Egypt; 4 Department of Chest Diseases, Faculty of Medicine, Ain-Shams University, Cairo, Egypt; 5 Department of Cardiology, Faculty of Medicine, Ain-Shams University, Cairo, Egypt; 6 Department of Medical Biochemistry and Molecular Biology, Faculty of Medicine, Ain Shams University, Cairo, Egypt; 7 Faculty of Medicine, Dar Al Uloom University, Riyadh, Saudi Arabia; 8 Department of Geriatrics Medicine and Gerontology, Faculty of Medicine, Ain Shams University, Cairo, Egypt; 9 Department of Public Health, Faculty of Applied Medical Sciences, Al-Baha University, Al-Baha, KSA Saudi Arabia; 10 Department of Laboratory Medicine, Faculty of Applied Medical Sciences, Al-Baha University, Al-Baha, KSA Saudi Arabia

**Keywords:** COVID-19, Cytokine storm, MiR-155, Mortality, Severity, Biomarkers, Diseases, Immunology, Medical research

## Abstract

The pathogenesis of the coronavirus disease 2019 (COVID-19) involves a complex group of cytokines and is significantly influenced by genetic and epigenetic factors. The current study aims to assess the association of COVID-19 severity and mortality with the relative expression of MicroRNA-155 (miR-155) and its association with serum levels of interleukin (IL)-6, IL-10, and their derived ratio. This pilot case-control study included 75 COVID-19 patients and 25 healthy controls. Serum levels of cytokines were analyzed by enzyme-linked immunosorbent assay (ELISA), and assessment of miR-155 relative expression level was done using quantitative real-time PCR in all participants. We found that IL-6, IL-10, IL-6/IL-10 ratio, and miR-155 expression levels were significantly higher in COVID-19 patients than in healthy controls (*p*-values < 0.001). The expression level of miR-155 showed significant differences between all severity categories of COVID-19, reaching its highest levels in the severe group. It was also significantly higher in ICU-admitted patients (*p*-value < 0.001) and those who died during their hospital stay (*p*-value = 0.001). It showed a significant negative correlation with serum IL-10 (*r*=-0.249, *p*-value = 0.031) and a significant positive correlation with IL-6/IL-10 ratio (*r* = 0.234, *p*-value = 0.043). It was also the only independent risk factor for COVID-19 severity by regression analysis and the best predictor for COVID-19 severity by ROC curve analysis. This study reported elevation of cytokines and miR-155 expression in COVID-19 patients. The level of miR-155 expression was associated with COVID-19 severity, mortality, and ICU admission, indicating its potential utility as a biomarker for monitoring the progression of COVID-19 and guiding patient follow-up.

## Introduction

Three serious pandemics, the coronavirus disease 2019 (COVID-19), the Middle East respiratory disease (MERS) in 2012, and the severe acute respiratory syndrome (SARS) in 2003, were linked to coronaviruses^1,2^. The severe acute respiratory syndrome coronavirus 2 (SARS-CoV-2) caused the highly contagious COVID-19 infection^2^. Despite quarantine regulations, the virus spread fast over the world and there were no specific, effective treatments^3^. COVID-19 has caused a global health crisis with wide variations in disease severity, ranging from asymptomatic infection to critical illness and death. Vaccination programs have played an essential role in reducing disease spread and severity^4^. While most COVID-19 patients have mild symptoms and a favorable prognosis, some may experience serious fatal consequences due to hyperinflammation caused by cytokine storms^5^. Excessive cytokine production during the cytokine storm can trigger an uncontrolled immune response and result in lung tissue damage and multiorgan failure^6^. Therefore, to avoid and treat the cytokine storm early and lower mortality in patients with severe COVID-19, it is important to understand the underlying mechanisms and predisposing factors for it.

Moreover, the outcome of COVID-19 infection is significantly influenced by genetic and epigenetic factors. MicroRNAs, or miRNAs, are a significant component of the epigenetic factors that control many immune cell and viral behaviors^7^. Of all the miRNAs, miRNA-155 (miR-155) which has been reported to have significant regulatory effects on the immune system and inflammatory response following viral infections^8,9^. Many previous studies demonstrated that following viral infection, miR-155 rises with the degree of lung inflammation, disease severity, and mortality rate^10,11^. In addition, the inhibition of this miRNA was reported to reduce lung inflammation and mortality in models of respiratory viral infections^9^.

The current study aims to assess the association of COVID-19 severity and mortality with the relative expression of miR-155 and its association with serum levels of the pro-inflammatory cytokine interleukin (IL)−6, the anti-inflammatory cytokine IL-10, and their derived ratio as indicators for the cytokine storm.

## Methodology

### Study settings and subjects

The current pilot case-control study was conducted on 75 confirmed COVID-19 patients recruited from Ain Shams University Hospitals from September 2021 to August 2022. COVID-19 infection was routinely confirmed in all patients by the hospital’s Clinical Pathology Department using real-time reverse transcription-polymerase chain reaction (RT-PCR) (VIASURE, Bio-Rad) from respiratory specimens, according to WHO guidelines^12^. The study excluded participants with immunological disorders and other active illnesses, those undergoing chemotherapy or radiation therapy, and pregnant women. Twenty-five age- and sex-matched healthy controls were also included. They were selected from relatives of the patients, had no history of COVID-19 infection, and were clinically free from acute or chronic illnesses. Based on the severity of their disease, COVID-19 participants were divided equally (25 participants in each group) into three groups (mild, moderate, and severe) in accordance with the Ain Shams University Hospital Consensus Report for handling adult COVID-19 patients^13^. All COVID-19 participants were followed up for their outcome either in-hospital death, or discharge from the hospital and the duration of hospital stay was determined for each patient.

### Clinical evaluation and sample collection

On admission, a complete medical history, clinical examination, and radiological evaluation were performed on each subject. All blood samples for biomarker assessment were collected upon admission, before the initiation of any COVID-19–related treatment, to ensure baseline measurement and avoid potential drug-related bias.

### Assessment of IL-6 and IL-10

From a 3 ml serum sample that was withdrawn from each participant, serum levels of IL-6 and IL-10 were determined by enzyme-linked immunosorbent assay (ELISA) technique using commercially available human kits (BT Laboratory, Shanghai, China, Catalog Numbers: E0090Hu and E0102Hu, respectively). The sensitivity of the IL-6 kit was 1.03 pg/mL and the IL-10 kit was 2.59 pg/mL.

### Assessment of miR-155 relative expression

From a 3 mL whole blood sample on an EDTA vacutainer tube that was also withdrawn from each participant, miR-155 relative expression was assessed by PCR analysis. Within an hour of the EDTA sample collection, double centrifugation was carried out. After a first centrifugation that lasted for ten minutes at 1900 ×g, the resulting plasma supernatant was carefully transferred into a separate, sterile tube and centrifuged again for ten minutes at 16,000 ×g. Until the extraction of miRNA, plasma was kept frozen at −80 °C. Plasma was selected instead of serum to avoid artificial contamination of circulating miRNAs that can occur due to the release of platelet-derived and intracellular miRNAs during the coagulation process.

#### MiRNA extraction, reverse transcription (RT) reaction

By the instructions of miRNeasy serum/plasma Kit (Qiagen, Germany), extraction of total miRNA from 200 µL plasma was performed. The concentration of the extracted total RNA was assesed by Qubit RNA HS assay kit on Qubit 3.0 fluorometer (Thermofisher Scientific, USA). A 5 µL of the RNA extract, containing 1–10 ng was used in the RT reaction mixture as recommended by the manufacturer. The extracted miRNA was used for further complementary DNA (cDNA) reaction using a specific stem-loop RT-primer from the MicroRNA TaqMan^®^ assay and TaqMan^®^ MicroRNA Reverse Transcription Kit (Applied Biosystems, USA) according to the manufacturer’s instructions. The prepared 10 µL RT-reaction was then incubated for 30 min at 16 °C and 42 °C, respectively, followed by a stop reaction of 5 min at 85 °C.

#### Real-Time quantitative polymerase chain reaction (RT-qPCR)

The resultant cDNA was then amplified using TaqMan Universal Master Mix and MicroRNA TaqMan^®^ assay (ipu-miR-155 assay, cat no. 4440886, test ID: 467534_mat) and the U6 snRNA (cat no. 4427975, test ID: 001973). In brief, five microliters of RT products were used as templates in 20 µL PCR reactions containing assays of miR-155 and U6 snRNA according to the manufacturer’s handbook. The amplification mix was incubated on the Applied Biosystems 7500 Real-Time PCR Detection System with the following conditions: initial activation at 95 °C for 10 min followed by 35 cycles of denaturation at 95 °C for 15 s and annealing/extension at 60 °C for 60 s.

MiR-155 expression was normalized to U6 snRNA expression, which was selected as an endogenous control based on its consistent detection and Ct stability across all study samples. Although U6 is a commonly used reference for cellular RNA, several previous studies have validated its applicability and stability in plasma miRNA profiling, including in COVID-19 patients and other inflammatory conditions^14–16^. Relative quantification in each sample was calculated using the delta-delta Ct (2^−ΔΔCT^) method.

### Statistical methods

Statistical presentation and analysis of the present study were conducted using the median and interquartile range (IQR) for quantitative data and number and percentage for qualitative data by the Statistical Package for Social Science (SPSS) V26 (IBM Corp., USA). The normality of quantitative data was assessed using the Shapiro–Wilk test. As most variables were not normally distributed, nonparametric tests were applied. Comparisons were done using the Mann-Whitney, Kruskal-Wallis, and Chi-square tests. For multiple group comparisons, pairwise Mann–Whitney tests were used as post-hoc analyses following the Kruskal–Wallis test. Spearman’s coefficient was used for the detection of the correlation between variables. Risk factors for COVID-19 severity were identified using logistic regression analysis by odds ratios (ORs) and 95% confidence intervals (CIs). The predicting ability for COVID-19 severity and mortality was evaluated by the receiver operating characteristic (ROC) curve analysis. Significant *p*-values are < 0.05.

## Results

The current study included 75 COVID-19 patients, 42 (56%) males and 33 (44%) females. Their median (IQR) age was 64 (54–69.5.5) years and ranged from 29 to 88 years. Of them, 38 (50.67%) required intensive care unit (ICU) admission. Fifty-four (72%) patients were discharged alive, while 21 (28%) patients died during their hospital stay. Hypertension (52/75, 69.33%) and diabetes (38/75, 50.67%) were the most associated comorbid conditions. Regarding chest CT scan findings of the included COVID-19 patients, 6.67% (5/75) were free and 93.33% (70/75) showed bilateral ground-glass opacity (GGO). The median (IQR) of hospital stay of the COVID-19 patients was 12 (8 −15.5) days and ranged from 3 to 36 days.

Compared to the control group, COVID-19 patients showed significantly higher levels of IL-6, IL-10, IL6/IL-10 ratio, and miR-155 relative expression (*p*-values < 0.001) (Table 1).

**Table 1:** Comparison of levels of IL-6, IL-10, IL-6/IL-10 ratio, and miR-155 relative expression between all COVID-19 patients (*n* = 75) and controls (*n* = 25).

	Controls				COVID-19				Mann-Whitney test	
	Median	IQR			Median	IQR			Z	*p*-value
IL-6 (pg/mL)	4.00	2.50	-	5.00	75.00	51.00	-	125.00	7.461	< 0.001*
IL-10 (pg/mL)	5.50	5.00	-	5.80	45.00	24.50	-	65.00	7.467	< 0.001*
IL-6/IL-10 ratio	0.71	0.50	-	0.95	1.83	0.96	-	3.75	4.418	< 0.001*
MiR-155-fold expression	2.09	0.28	-	2.24	8.96	6.34	-	17.33	7.262	< 0.001*

Abbreviations: IL: Interleukin.

*p-value < 0.05 is significant.

When laboratory data were compared according to COVID-19 severity, IL-6, IL-6/IL-10 ratio, and miR-155 expression showed the highest values in the severe group while serum IL-10 reached its lowest level in the severe group. Serum IL-6 and IL-10 showed significant differences between mild and severe patients only (*p*-values = 0.003, < 0.001, respectively). IL-6/IL-10 ratio showed significant differences between mild and severe patients (*p*-value = 0.003) and between moderate and severe patients (*p*-value < 0.001). On the other hand, miR-155 expression showed significant differences between all the severity groups with *p*-values of < 0.001 (Table 2).

**Table 2:** Comparison of laboratory data of COVID-19 patients according to disease severity.

	Mild (*n* = 25)				Moderate (*n* = 25)				Severe (*n* = 25)				Kruskal-Wallis test		Mann-Whitney test			
	Median	IQR			Median	IQR			Median	IQR			X^2^	*p*-value	Mild vs. Moderate		Mild vs. Severe	Moderate vs. Severe
IL-6 (pg/mL)	52.00	30.00	-	90.00	70.00	51.00	-	130.00	90.20	75.00	-	162.50	8.870	0.012*	0.111		0.003*	0.196
IL-10 (pg/mL)	65.00	42.00	-	88.00	45.00	32.00	-	60.00	30.00	20.00	-	45.00	13.946	0.001*	0.066		< 0.001*	0.053
IL-6/IL-10 ratio	1.44	0.58	-	2.89	1.24	0.67	-	2.33	3.36	1.88	-	6.25	14.323	0.001*	0.727		0.003*	< 0.001*
MiR-155-fold expression	5.52	3.90	-	6.79	9.61	7.80	-	12.68	25.35	17.93	-	35.85	50.472	< 0.001*	< 0.001*		< 0.001*	< 0.001*

Abbreviations: IL: Interleukin.

*p-value < 0.05 is significant.

Compared to the cured and discharged COVID-19 patients (*n* = 54), expression levels of miR-155 and IL-6/IL-10 ratio were significantly higher in patients who died (*n* = 21) (*p*-values = 0.001), while serum level of IL-10 was significantly lower (*p*-value < 0.001). On the other hand, serum IL-6 did not show any significant differences between cured patients and those who died (*p*-value = 0.240) (Table 3).

**Table 3:** Laboratory data compared according to COVID-19 patients’ outcome.

	Cured and discharged (*n* = 54)				Died (*n* = 21)				Mann-Whitney test	
	Median	IQR			Median	IQR			Z	*p*-value
IL-6 (pg/mL)	70.00	51.00	-	100.80	90.20	70.00	-	140.00	1.176	0.240
IL-10 (pg/mL)	55.50	40.00	-	72.00	20.00	16.00	-	32.70	4.629	< 0.001*
IL-6/IL-10 ratio	1.39	0.70	-	2.78	3.89	2.50	-	6.25	3.322	0.001*
MiR-155-fold expression	7.80	5.52	-	12.68	17.93	9.61	-	27.17	3.372	0.001*

Abbreviations: IL: Interleukin.

*p-value < 0.05 is significant.

When ICU-admitted patients (*n* = 38) were compared to those who were not admitted to the ICU (*n* = 37), serum IL-6 and IL-6/IL-10 ratio did not show any significant differences (*p*-value = 0.937, 0.330, respectively). On the other hand, serum IL-10 significantly decreased in ICU-admitted patients (*p*-value = 0.024), while miR-155 expression significantly increased (*p*-value < 0.001) (Table 4).

**Table 4:** Laboratory data compared according to ICU admission of COVID-19 patients.

	No ICU (*n* = 37)				ICU (*n* = 38)				Mann-Whitney test	
	Median	IQR			Median	IQR			Z	*p*-value
IL-6 (pg/mL)	70.00	51.00	-	125.00	85.00	51.00	-	125.00	0.080	0.937
IL-10 (pg/mL)	55.00	33.00	-	70.00	34.50	20.00	-	56.00	2.254	0.024*
IL-6/IL-10 ratio	1.82	0.85	-	2.80	1.97	1.04	-	5.00	0.975	0.330
MiR-155-fold expression	6.79	5.15	-	8.36	16.73	9.61	-	31.21	5.197	< 0.001*

Abbreviations: ICU: Intensive care unit; IL: Interleukin.

*p-value < 0.05 is significant.

**Fig. 1:**
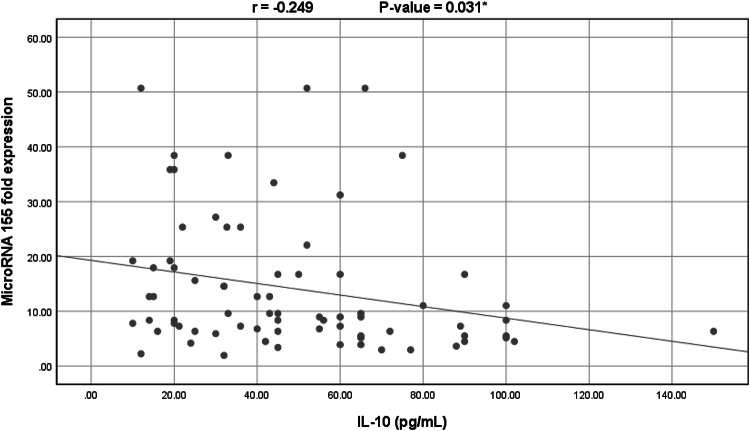
Significant negative correlation between serum IL-10 levels and miR-155 expression levels in all COVID-19 patients (*n* = 75) (Spearman’s *r* = − 0.249, *p*-value = 0.031).

**Fig. 2:**
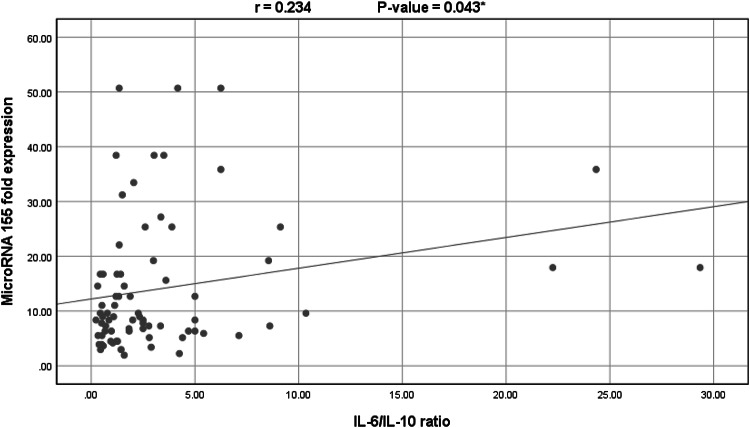
Significant positive correlation between the IL-6/IL-10 ratio and miR-155 expression levels in all COVID-19 patients (*n* = 75) (Spearman’s *r* = 0.234, *p*-value = 0.043).

Spearman correlation study was performed and showed that miR-155 expression level was significantly negatively correlated with IL-10 (*r*=−0.249, *p*-value = 0.031) and significantly positively correlated with IL-6/IL-10 ratio (*r* = 0.234, *p*-value = 0.043) but showed no significant correlation with serum IL-6 levels (*r* = 0.130, *p*-value = 0.268). No other statistically significant correlations were observed *(p*-values > 0.05) (Figs. 1 and 2).

When logistic regression analysis was performed after adjustment, miR-155 expression level remained a significant (*p*-value = 0.014) independent risk factor for severe COVID-19, with an OR of 2.537 and a 95% CI of 1.090–4.169 (Table 5).

**Table 5:** Regression analysis for risk factors of severe COVID-19.

Severity	Odd’s ratio	95% C.I. for Odd’s ratio			*p*-value
IL-6 (pg/mL)	0.989	0.946	-	1.034	0.632
IL-10 (pg/mL)	0.963	0.865	-	1.072	0.491
IL-6/IL-10 ratio	2.093	0.425	-	10.315	0.364
MiR-155-fold expression	2.537	1.090	-	4.169	0.014*

Abbreviations: IL: Interleukin.

*p-value < 0.05 is significant.

**Fig. 3:**
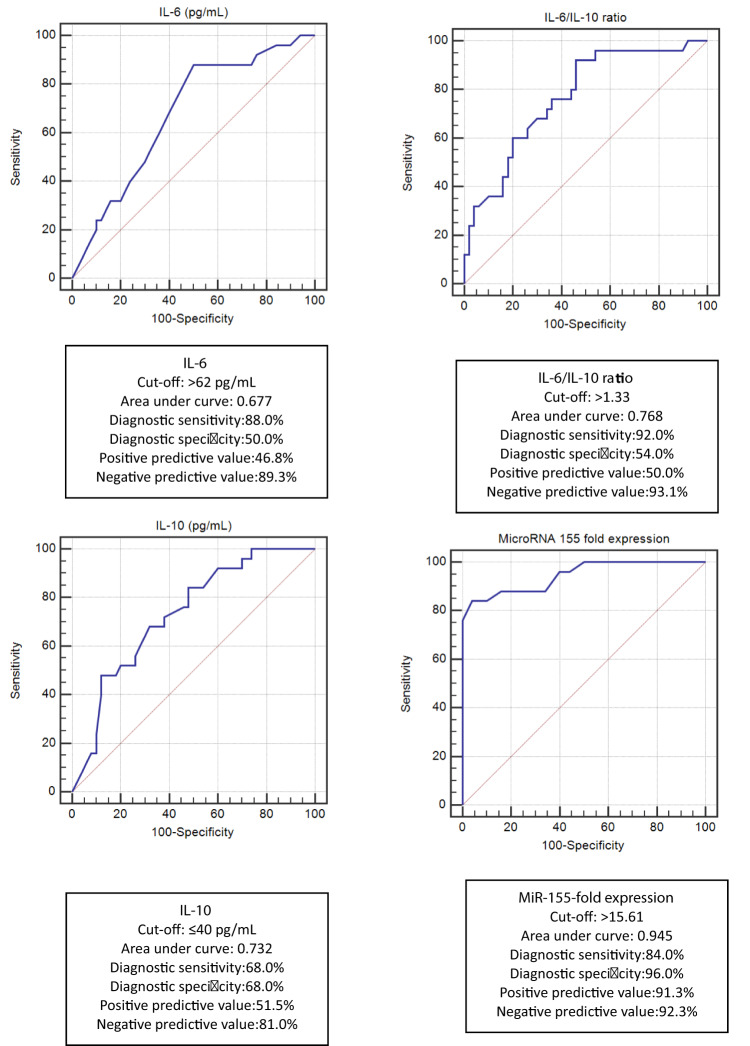
Receiver Operating Characteristic (ROC) curve analyses for prediction of COVID-19 severity in all COVID-19 patients (*n* = 75).

IL-6, IL-10, IL-6/IL-10 ratio, and miR-155 expression were evaluated by ROC curve analyses for prediction ability for COVID-19 severity and mortality. At a cut-off of > 15.61, miR-155 expression was the best predictor for COVID-19 severity. Its area under the curve (AUC) was 0.945, with the best diagnostic specificity (96%) and positive predictive value (91.3%) (Fig. 3).

**Fig. 4:**
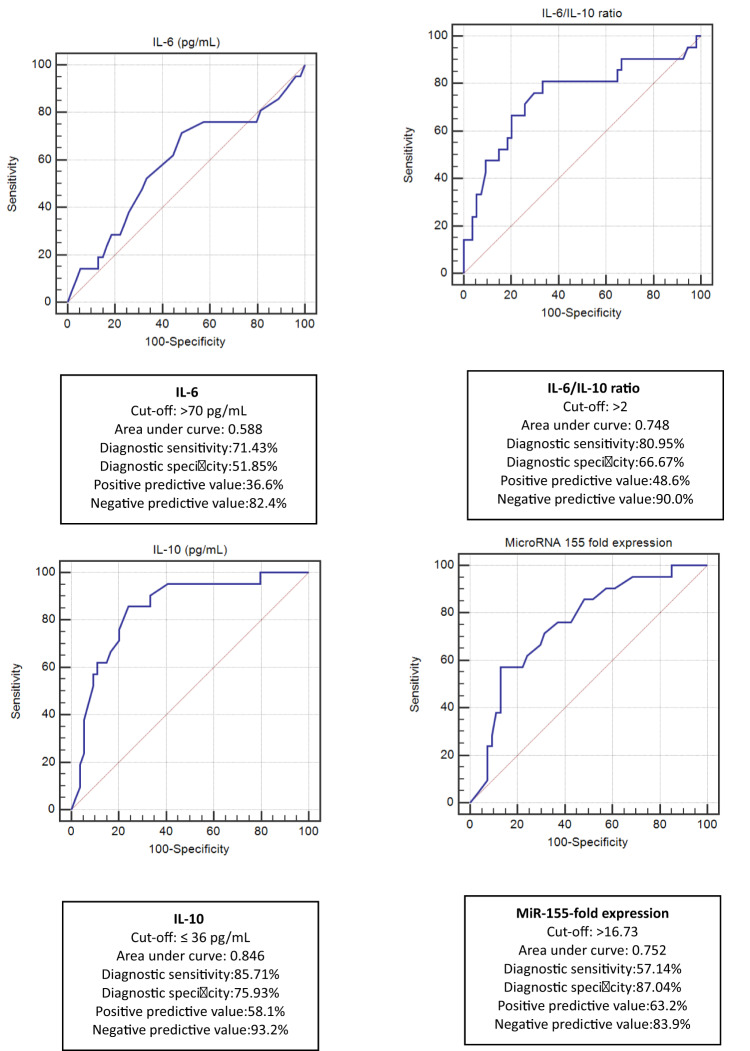
Receiver Operating Characteristic (ROC) curves for prediction of COVID-19 mortality in all COVID-19 patients (*n* = 75).

On the other hand, serum IL-10 at a cut-off of ≤ 36 pg/mL was the best predictor for COVID-19 mortality. Its AUC was 0.846, with the best diagnostic sensitivity (85.71%) and negative predictive value (93.2%) (Fig. 4).

## Discussion

The current study aimed to spotlight the interplay between miR-155 expression levels and inflammatory cytokines IL-6 and IL-10 with their derived ratio in the context of COVID-19 severity and mortality. We found that miR-155 expression levels were significantly higher in COVID-19 patients than in healthy controls. Furthermore, miR-155 was the only marker that showed significant differences between all severity categories of COVID-19, reaching its highest levels in the severe group. It was also significantly higher in ICU-admitted patients and those who died during their hospital stay. Also, it was the only independent risk factor for COVID-19 severity by regression analysis. These findings underline the potential clinical value of miR-155 as a non-invasive biomarker for disease progression and outcome prediction in COVID-19.

MiR-155 is encoded by the MIRHG155 gene, originally identified as the B-cell Integration Cluster (BIC) gene, on chromosome 21^17,18^. It has been extensively studied in relation to host-pathogen dynamics in human viral infections and has been linked to immune modulation^19^. Expression of miR-155 occurs in B cells, T cells, dendritic cells, and macrophages and is essential for cell-mediated immune responses^20^. In response to inflammatory stimuli, miR-155 expression was reported to be elevated within hours and is associated with pro-inflammatory transcription^20^.

Our results extend this understanding by demonstrating that circulating miR-155 levels are not only elevated in COVID-19 patients but also progressively increase with disease severity, reflecting its potential role as a molecular marker of hyperinflammation.

Although our study does not provide direct mechanistic evidence, previous studies have suggested that miR-155 may influence inflammatory pathways, such as SHIP1/SOCS1 and NF-κB signaling, which regulate cytokine production. For instance, miR-155 has been shown to promote IL-6 production by downregulating SHIP1 and SOCS1, negative regulators of the JAK/STAT signaling pathway^18,20^, and to potentiate inflammation by promoting NF-κB activity in macrophages through Toll-like receptor signaling^21^. Moreover, several previous studies reported that knocking out of miR-155 in mice has resulted in a decreased lung inflammatory response with fast recovery^22,23^. Furthermore, Koranteng et al., 2004^24^ reported that the inflammatory response could be effectively reduced by inhibiting the miR-155/NF-kB axis. These mechanisms, reported in experimental models, may partly explain the observed associations in our study and should be further investigated in future research.

In accordance with our results, Haroun et al., 2022^18^demonstrated that the miR-155 expression level was higher in their included COVID-19 patients than in controls and in severe COVID-19 cases than in the moderate ones. They also reported that miR-155 expression increased in non-survivals compared to survivals. Similar results were reported by Gedikbasi et al.^20^, and Keikha et al., 2023^25^. They found that the relative expression of miR-155 was significantly increased with the increase in COVID-19 severity. Furthermore, Garg et al., 2021^26^, reported that miR-155 expression levels were significantly higher in COVID-19 patients than in healthy controls and patients with influenza-associated acute respiratory distress syndrome.

By confirming and extending these observations, our data reinforce the reproducibility of miR-155 upregulation in COVID-19 and further establish its association with clinical severity and mortality, highlighting its prognostic relevance.

On the other hand, Kassif-Lerner et al., 2022^27^ found a significant decrease in miR-155 expression levels in COVID-19 patients than controls as mild patients had 2.5-fold less miR-155 and severe patients had 5-fold less miR-155 with no significant difference in miR-155 expression between mild and severe COVID-19 patients. They also reported that patients who died had significantly less miR-155 than patients who survived. These discrepancies from our findings may not only be attributed to their relatively small sample size (37 patients) but also to several methodological and biological factors. Differences in patient demographics and clinical characteristics, including age, comorbidities, and disease stage at sampling, could have influenced miRNA expression. Additionally, variations in the timing of sample collection relative to infection onset, differences in RNA extraction protocols, and the choice of internal reference or normalization strategy for miRNA quantification may have contributed to the contrasting results. Together, these factors highlight the complexity of comparing miRNA expression studies across heterogeneous cohorts.

Additionally, miR-155 expression in the current study was the best predictor for COVID-19 severity with a diagnostic sensitivity of 84% and diagnostic specificity of 96% at a cut-off of > 15.61. This high discriminative power suggests that miR-155 could be clinically valuable for early risk stratification of COVID-19 patients. Haroun et al., 2022^18^ found that the AUC for miR-155 expression was 0.75 with 76% sensitivity and specificity for the prediction of COVID-19 severity. Also, Garg et al., 2021^26^ found that miR-155 had a strong discriminative power between COVID-19 and influenza-associated acute respiratory distress syndrome with an AUC of 1.00. Our results thus bridge the gap between studies, positioning miR-155 as a promising yet context-dependent biomarker that warrants validation in larger, multicentric cohorts.

Due to interindividual variability in serum IL-6 levels according to the time of the same day and variations in the associated immune-metabolic comorbidities like obesity, we evaluated the IL-6/IL-10 ratio since serum IL-6 levels alone may not be an accurate indication of COVID-associated hyperinflammation^28^. McElvaney et al., 2020^28^ used the changes in the IL-6/IL-10 ratio to derive a scoring system and found that each 1-point increase in the score was associated with an increased risk for a more severe outcome by 5.6 times. In the current study, we found that serum IL-6, IL-10, and IL-6/IL-10 ratio were significantly higher in COVID-19 patients than controls. Serum IL-6 and IL-6/IL-10 ratio reached their highest levels in the severe group, while serum IL-10 reached the lowest levels in the severe group.

The pathogenesis of COVID-19 involves a complex group of mediators, including IL-6 and IL-10^29^. Several studies reported elevation of serum IL-6 and IL-10 in COVID-19 patients, and this elevation was dramatic with severe COVID-19 due to hyperactivation of the humoral immune system^29–32^. The discrepancy between our study and the other research regarding IL-10 levels could be attributed to the differences in sample sizes and techniques used in cytokine identification with different sensitivities. Also, we did not study the molecular background and regulatory mechanisms on the cellular level that may impact this cytokines’ expression level.

Moreover, in the current study, miR-155 expression had a negative correlation with serum IL-10 and a positive correlation with IL-6/IL-10 ratio. However, these correlations were weak despite reaching statistical significance and therefore should be interpreted with caution. They suggest a potential but not definitive relationship between miR-155 and cytokine balance in COVID-19. In addition, miR-155 expression showed no significant correlation with serum IL-6 alone. According to Mahesh et al., 2019^33^, IL-10 can suppress the transcription factor Ets2, which reduces miR-155 expression. Additionally, it has been found that inhibiting miR-155 reduces IL-6 expression by 31%^34^. This is because miR-155 induces the IL-6 signaling pathway through the transcription factor C/EBPβ, which in turn stimulates the production of the IL-6 gene^35^. This study is the first to evaluate the association between miR-155 expression levels and the IL-6/IL-10 ratio as a more stable marker for COVID-associated hyperinflammation.

However, several limitations should be acknowledged. First, the sample size was limited, particularly after subdividing COVID-19 patients based on disease severity, which may have impacted the statistical power of our findings. Additionally, the study did not account for comorbid conditions or other potential confounding factors that could have influenced the results. Furthermore, as this was a single-center study conducted in Egypt, the findings may not be fully generalizable to other populations or healthcare settings. Another limitation is the limited number of cytokines analyzed, as only IL-6 and IL-10 were measured, which may not fully reflect the complexity of the immune-inflammatory response in COVID-19. Moreover, the study lacked long-term follow-up of discharged patients, which would have provided valuable insights into the sustained impact of miR-155 and cytokine levels. The limited sample size also imposed certain statistical constraints, particularly after stratification by disease severity.

Future studies are recommended to include larger, multi-center, and more ethnically diverse cohorts to enhance the generalizability of the findings. Incorporating a broader panel of cytokines and microRNAs, along with detailed clinical and immunological profiling, would provide a more comprehensive understanding of the immune-inflammatory landscape in COVID-19. Longitudinal studies with extended follow-up are also encouraged to evaluate the prognostic and therapeutic potential of miR-155 and related biomarkers over time. Moreover, adjustment for common comorbidities and treatment regimens in multivariate models will help clarify their independent contributions to disease severity and outcomes.

## Conclusions

In conclusion, our study contributes to the growing evidence highlighting the elevation of inflammatory cytokines with their derived ratio (IL-6/IL-10) and miR-155 expression in COVID-19 patients. Importantly, our findings suggest that miR-155 expression holds significant prognostic value being associated with COVID-19 severity, mortality, and ICU admission, indicating its potential utility as a biomarker for monitoring the progression of COVID-19 and guiding patient follow-up. The prognostic relevance of miR-155 underscores its potential role in improving clinical outcomes by enabling more precise risk stratification and targeted interventions in COVID-19 management. Taken together, our clinical findings, in line with previous experimental reports, indicate a possible role of miR-155 in modulating inflammatory responses. However, this remains a hypothesis-generating observation that requires validation through mechanistic studies. These insights pave the way for further research into the interplay between miR-155 and inflammatory cytokines in the context of COVID-19, with implications for both clinical practice and therapeutic development.

## Data Availability

All the data needed to support the current findings will be available upon request.

## Abbreviations

COVID-19: Coronavirus disease 2019
MERS: The Middle East respiratory disease
SARS: The severe acute respiratory syndrome
SARS-CoV-2: The severe acute respiratory syndrome coronavirus 2
IL: Interleukin
RT-PCR: Real-time reverse transcription-polymerase-chain reaction
ELISA: Enzyme-linked immunosorbent assay
ICU: Intensive care unit
GGO: Ground-glass opacity

## References

[1] Peiris, M. & Poon, L. L. M. Severe Acute Respiratory Syndrome (SARS) and Middle East Respiratory Syndrome (MERS) (*Coronaviridae*). Encyclopedia of Virology. :814–24. (2021). 10.1016/B978-0-12-814515-9.00138-7. Epub 2021 Mar 1. PMCID: PMC7837069.

[2] Gorbalenya, A. et al. The species severe acute respiratory syndrome-related coronavirus: classifying 2019-nCoV and naming it SARS-CoV-2. *Nat. Microbiol.* **5** (4), 536–544. 10.1038/s41564-020-0695-z (2020). Epub 2020 Mar 2. PMID: 32123347; PMCID: PMC7095448. 32123347 10.1038/s41564-020-0695-z PMC7095448

[3] Jiang, Y. et al. Cytokine storm in COVID-19: from viral infection to immune responses, diagnosis and therapy. *Int. J. Biol. Sci.* **18** (2), 459–472. 10.7150/ijbs.59272 (2022). PMID: 35002503; PMCID: PMC8741849. 35002503 10.7150/ijbs.59272 PMC8741849

[4] Kandeel, A. et al. COVID-19 vaccination coverage in egypt: a large-scale National survey - to help achieving vaccination target, March-May, 2022. *BMC Public. Health*. **23** (1), 397. 10.1186/s12889-023-15283-w (2023). PMID: 36849954; PMCID: PMC9969364. 36849954 10.1186/s12889-023-15283-w PMC9969364

[5] Elessawy, S. M., Shehab, A., Soliman, D. A., Eldeeb, M. A. & Taha, S. I. Interferon-Induced Transmembrane Protein-3 Rs12252-G Variant Increases COVID-19 Mortality Potential in Egyptian Population. Viral Immunol. 37(4):186–193. (2024). 10.1089/vim.2024.0015. PMID: 38717821. 10.1089/vim.2024.0015 38717821

[6] Jose, R. J. & Manuel, A. COVID-19 cytokine storm: the interplay between inflammation and coagulation. Lancet Respir Med. ;8(6):e46-e47. doi: 10.1016/S2213-2600(20)30216-2. Epub 2020 Apr 27. PMID: 32353251; PMCID: PMC7185942. (2020). 10.1016/S2213-2600(20)30216-2 PMC7185942 32353251

[7] Asadpour-Behzadi, A., Kariminik, A. & Kheirkhah, B. MicroRNA-155 is a main part of proinflammatory puzzle during severe coronavirus disease 2019 (COVID-19). Allergol Immunopathol (Madr). 51(2):115–119. (2023). 10.15586/aei.v51i2.698. PMID: 36916095. 10.15586/aei.v51i2.698 36916095

[8] Lind, E. F. & Ohashi, P. S. Mir-155, a central modulator of T-cell responses. Eur J Immunol. ;44(1):11 – 5. (2014). 10.1002/eji.201343962. PMID: 24571026. 10.1002/eji.201343962 24571026

[9] Soltani-Zangbar, M. S. et al. SARS-CoV2 infection induce miR-155 expression and skewed Th17/Treg balance by changing SOCS1 level: A clinical study. *Cytokine* **169**, 156248 (2023). Epub 2023 Jun 8. PMID: 37307689; PMCID: PMC10247889. 37307689 10.1016/j.cyto.2023.156248 PMC10247889

[10] Arroyo, M. et al. Airway mir-155 responses are associated with TH1 cytokine polarization in young children with viral respiratory infections. *PLoS One*. **15** (5), e0233352. 10.1371/journal.pone.0233352 (2020). PMID: 32442188; PMCID: PMC7244143. 32442188 10.1371/journal.pone.0233352 PMC7244143

[11] Natekar, J. P., Rothan, H. A., Arora, K., Strate, P. G. & Kumar, M. Cellular microRNA-155 regulates virus-Induced inflammatory response and protects against lethal West nile virus infection. *Viruses* **12** (1), 9. 10.3390/v12010009 (2019). PMID: 31861621; PMCID: PMC7019255. 31861621 10.3390/v12010009 PMC7019255

[12] World Health Organization. Coronavirus disease (COVID-19) Weekly Epidemiological Update and Weekly Operational Update. (2021). Available from: https://www.who.int/emergencies/diseases/novel-coronavirus-2019/situationreports [Last accessed November 22, 2021].

[13] Abdelfattah, E. et al. Hospital response to COVID-19 A consensus report on Ain Shams university hospital strategy. *ScienceOpen Preprints*. 10.14293/S2199-1006.1.SOR-.PPD4QZX.v1 (2020).

[14] Belete, M. A. et al. The potential of Circulating MicroRNAs as novel diagnostic biomarkers of COVID-19: a systematic review and meta-analysis. *BMC Infect. Dis.* **24** (1), 1011. 10.1186/s12879-024-09915-8 (2024). PMID: 39300343; PMCID: PMC11414062. 39300343 10.1186/s12879-024-09915-8 PMC11414062

[15] Pollet, K. et al. Host MiRNAs as biomarkers of SARS-CoV-2 infection: a critical review. *Sens. Diagnostics*. **2** (1), 12–35. 10.1039/d2sd00140c (2023).

[16] de Santana Silva, Í. T. S. et al. Identification of SnRNA U6 as an endogenous reference gene for normalization of MiRNA expression data in COVID-19 patients. *Sci. Rep.* **15** (1), 26636. 10.1038/s41598-025-04062-9 (2025). PMID: 40695852; PMCID: PMC12284167. 40695852 10.1038/s41598-025-04062-9 PMC12284167

[17] Hu, J. et al. miR-155: an important role in inflammation response. *J. Immunol. Res.* **2022**, 7437281. 10.1155/2022/7437281 (2022). PMID: 35434143; PMCID: PMC9007653. 35434143 10.1155/2022/7437281 PMC9007653

[18] Haroun, R. A. et al. Circulating plasma miR-155 is a potential biomarker for the detection of SARS-CoV-2 infection. *Pathology* **54** (1), 104–110. 10.1016/j.pathol.2021.09.006 (2022). Epub 2021 Nov 6. PMID: 34838331; PMCID: PMC8570980. 34838331 10.1016/j.pathol.2021.09.006 PMC8570980

[19] Li, Y. & Kowdley, K. V. MicroRNAs in common human diseases. *Genomics Proteom. Bioinf.* **10** (5), 246–253. 10.1016/j.gpb.2012.07.005 (2012). Epub 2012 Sep 29. PMID: 23200134; PMCID: PMC3611977. 10.1016/j.gpb.2012.07.005 PMC3611977 23200134

[20] Gedikbasi, A. et al. The effect of host MiRNAs on prognosis in COVID-19: miRNA-155 May promote severity via targeting suppressor of cytokine signaling 1 (*SOCS1*) gene. *Genes (Basel)*. **13** (7), 1146. 10.3390/genes13071146 (2022). PMID: 35885930; PMCID: PMC9320261. 35885930 10.3390/genes13071146 PMC9320261

[21] Mann, M. et al. An NF-κB-microRNA regulatory network tunes macrophage inflammatory responses. Nat Commun. 8(1):851. (2017). 10.1038/s41467-017-00972-z. Erratum in: Nat Commun. 2018; 9(1):3338. DOI: 10.1038/s41467-018-05720-5. PMID: 29021573; PMCID: PMC5636846. 10.1038/s41467-017-00972-z PMC5636846 29021573

[22] Pociask, D. A. et al. Epigenetic and transcriptomic regulation of lung repair during recovery from influenza infection. *Am. J. Pathol.* **187** (4), 851–863 (2017). Epub 2017 Feb 10. PMID: 28193481; PMCID: PMC5397680. 28193481 10.1016/j.ajpath.2016.12.012 PMC5397680

[23] De Smet, E. G. et al. The role of miR-155 in cigarette smoke-induced pulmonary inflammation and COPD. *Mucosal Immunol.* **13** (3), 423–436. 10.1038/s41385-019-0241-6 (2020). Epub 2019 Dec 9. PMID: 31819170. 31819170 10.1038/s41385-019-0241-6

[24] Koranteng, R. D. et al. Differential regulation of mast cell cytokines by both dexamethasone and the p38 mitogen-activated protein kinase (MAPK) inhibitor SB203580. *Clin. Exp. Immunol.* **137** (1), 81–87. 10.1111/j.1365-2249.2004.02510.x (2004). PMID: 15196247; PMCID: PMC1809098. 15196247 10.1111/j.1365-2249.2004.02510.x PMC1809098

[25] Keikha, R., Hashemi-Shahri, S. M. & Jebali, A. The MiRNA neuroinflammatory biomarkers in COVID-19 patients with different severity of illness. *Neurologia* **38** (6), e41–e51. 10.1016/j.nrl.2021.06.005 (2023 Jul-Aug). Epub 2021 Jul 16. PMID: 34305233; PMCID: PMC8282440. 10.1016/j.nrl.2021.06.005 PMC8282440 34305233

[26] Garg, A. et al. Circulating cardiovascular MicroRNAs in critically ill COVID-19 patients. *Eur. J. Heart Fail.* **23** (3), 468–475 (2021). Epub 2021 Mar 5. PMID: 33421274; PMCID: PMC8014268. 33421274 10.1002/ejhf.2096 PMC8014268

[27] Kassif-Lerner, R. et al. miR-155: A potential biomarker for predicting mortality in COVID-19 patients. *J. Pers. Med.* **12** (2), 324. 10.3390/jpm12020324 (2022). PMID: 35207812; PMCID: PMC8877479. 35207812 10.3390/jpm12020324 PMC8877479

[28] McElvaney, O. J. et al. A linear prognostic score based on the ratio of interleukin-6 to interleukin-10 predicts outcomes in COVID-19. EBioMedicine. 61: 103026. DOI: 10.1016/j.ebiom.2020.103026. Epub 2020 Oct 8. Erratum in: EBioMedicine. 2020; 62: 103116. (2020). 10.1016/j.ebiom.2020.103116. PMID: 33039714; PMCID: PMC7543971. 10.1016/j.ebiom.2020.103026 PMC7543971 33039714

[29] Han, H. et al. Profiling serum cytokines in COVID-19 patients reveals IL-6 and IL-10 are disease severity predictors. *Emerg. Microbes Infect.* **9** (1), 1123–1130 (2020). PMID: 32475230; PMCID: PMC7473317. 32475230 10.1080/22221751.2020.1770129 PMC7473317

[30] Zhao, Y. et al. Longitudinal COVID-19 profiling associates IL-1RA and IL-10 with disease severity and RANTES with mild disease. *JCI Insight*. **5** (13), e139834. 10.1172/jci.insight.139834 (2020). PMID: 32501293; PMCID: PMC7406242. 32501293 10.1172/jci.insight.139834 PMC7406242

[31] Wang, F. et al. The laboratory tests and host immunity of COVID-19 patients with different severity of illness. *JCI Insight*. **5** (10), e137799. 10.1172/jci.insight.137799 (2020). PMID: 32324595; PMCID: PMC7259533. 32324595 10.1172/jci.insight.137799 PMC7259533

[32] Coomes, E. A. & Haghbayan, H. Interleukin-6 in Covid-19: A systematic review and meta-analysis. *Rev. Med. Virol.* **30** (6), 1–9. 10.1002/rmv.2141 (2020). Epub 2020 Aug 26. PMID: 32845568; PMCID: PMC7460877. 32845568 10.1002/rmv.2141 PMC7460877

[33] Mahesh, G. & Biswas, R. MicroRNA-155: A master regulator of inflammation. *J. Interferon Cytokine Res.* **39** (6), 321–330. 10.1089/jir.2018.0155 (2019). Epub 2019 Mar 20. PMID: 30998423; PMCID: PMC6591773. 30998423 10.1089/jir.2018.0155 PMC6591773

[34] Pfeiffer, D., Roßmanith, E., Lang, I. & Falkenhagen, D. miR-146a, miR-146b, and miR-155 increase expression of IL-6 and IL-8 and support HSP10 in an in vitro sepsis model. *PLoS One*. **12** (6), e0179850. 10.1371/journal.pone.0179850 (2017). PMID: 28662100; PMCID: PMC5491059. 28662100 10.1371/journal.pone.0179850 PMC5491059

[35] Ceppi, M. et al. MicroRNA-155 modulates the interleukin-1 signaling pathway in activated human monocyte-derived dendritic cells. *Proc. Natl. Acad. Sci. U S A*. **106** (8), 2735–2740. 10.1073/pnas.0811073106 (2009). Epub 2009 Feb 4. PMID: 19193853; PMCID: PMC2650335. 19193853 10.1073/pnas.0811073106 PMC2650335

